# Noda-Like RNA Viruses Infecting *Caenorhabditis* Nematodes: Sympatry, Diversity, and Reassortment

**DOI:** 10.1128/JVI.01170-19

**Published:** 2019-10-15

**Authors:** Lise Frézal, Hyeim Jung, Stephen Tahan, David Wang, Marie-Anne Félix

**Affiliations:** a IBENS, Department of Biology, Ecole Normale Supérieure, CNRS, Inserm, PSL Research University, Paris, France; b Departments of Molecular Microbiology and Pathology & Immunology, Washington University in St. Louis School of Medicine, St. Louis, Missouri, USA; University of Texas Southwestern Medical Center

**Keywords:** *C. elegans*, *C. briggsae*, Santeuil virus, Le Blanc virus, Orsay virus, Mělník virus, nodavirus, genetic diversity, viral RNA reassortment, viral competition, coinfection

## Abstract

Three RNA viruses related to nodaviruses were previously described to naturally infect the nematode Caenorhabditis elegans and its relative, Caenorhabditis briggsae. Here, we report on a collection of more than 50 viral variants from wild-caught *Caenorhabditis.* We describe the discovery of a new related virus, the Mělník virus, infecting C. briggsae, which similarly infects intestinal cells. In France, a frequent pattern of coinfection of C. briggsae by the Santeuil virus and Le Blanc virus was observed at the level of an individual nematode and even a single cell. We do not find evidence of reassortment between the RNA1 and RNA2 molecules of Santeuil and Le Blanc viruses. However, by studying patterns of evolution of each virus, reassortments of RNA1 and RNA2 among variants of each virus were identified. We develop assays to test the relative infectivity and competitive ability of the viral variants and detect an interaction between host genotype and Santeuil virus genotype, such that the result depends on the host strain.

**IMPORTANCE** The roundworm Caenorhabditis elegans is a laboratory model organism in biology. We study natural populations of this small animal and its relative, C. briggsae, and the viruses that infect them. We previously discovered three RNA viruses related to nodaviruses and here describe a fourth one, called the Mělník virus. These viruses have a genome composed of two RNA molecules. We find that two viruses may infect the same animal and the same cell. The two RNA molecules may be exchanged between variants of a given viral species. We study the diversity of each viral species and devise an assay of their infectivity and competitive ability. Using this assay, we show that the outcome of the competition also depends on the host.

## Abstract (precis)

The roundworm Caenorhabditis elegans is a laboratory model organism in biology. We study natural populations of this small animal and its relative, C. briggsae, and the viruses that infect them. We previously discovered three RNA viruses related to nodaviruses and here describe a fourth one, called the Mělník virus. These viruses have a genome composed of two RNA molecules. We find that two viruses may infect the same animal and the same cell. The two RNA molecules may be exchanged between variants of a given viral species. We study the diversity of each viral species and devise an assay of their infectivity and competitive ability. Using this assay, we show that the outcome of the competition also depends on the host.

## INTRODUCTION

Three positive-strand RNA viruses related to nodaviruses were recently discovered to naturally infect the model organism Caenorhabditis elegans and its relative, Caenorhabditis briggsae (1, 2). The Orsay virus (ORV) was the first natural virus found to infect C. elegans. Its discovery enabled the definitive demonstration that the C. elegans RNA interference pathway is an important antiviral defense (1, 3). A genome-wide association study in C. elegans pointed to one major locus regulating the wide range of sensitivity to the Orsay virus of C. elegans wild isolates; this major locus corresponds to the *drh-1* gene, which encodes a viral sensor triggering both small RNA and transcriptional responses (3, 4). Laboratory forward genetics also revealed a SID-3/WASP pathway necessary for viral entry (5), while 3′-terminal uridylation of viral RNAs was shown to act in viral defense (6).

In addition to the Orsay virus found in C. elegans, two viruses of the same family were found in C. briggsae. The Santeuil virus (SANTV) was the first to be detected (1), followed by Le Blanc virus (LEBV) (2). These viruses were all shown to infect intestinal cells based on detection of viral RNAs and proteins as well as intestinal cell damage (1, 7), and they are horizontally transmitted (1). Their genome includes two RNA segments (1, 2, 8). The RNA1 segment encodes an RNA-dependent RNA polymerase (RdRp). The RNA2 segment encodes a viral capsid followed by an open reading frame (ORF), called delta. For ORV, translation of the capsid starts upstream of the first ATG and does not always end at the predicted stop codon due to ribosomal frameshifting leading to a fusion with the delta ORF (8). The ORV capsid structure has been solved (9). The full-length capsid-delta protein is present with low stoichiometry in ORV virions (8), with the delta part likely protruding from the surface of the particle and possibly mediating viral entry (9). In addition, free delta appears to mediate viral exit on the apical side of the intestinal cells (10). The receptors of these viruses on the surface of the intestinal cells are still unknown. The transcriptional responses upon infection by ORV in C. elegans and SANTV in C. briggsae are in part conserved and are partially shared with the response to the infection of intestinal cells by microsporidia (11–13).

Here, we aimed to collect *Caenorhabditis* viruses systematically in order to assess their diversity. *Caenorhabditis* nematodes are found in decomposing vegetal matter such as rotting fruits, stems, flowers, or compost (14–16). We thereby discovered a new C. briggsae virus, the Mělník virus (MELV), which we show is most closely related to SANTV. We established a collection of variants of each virus, especially of the two C. briggsae viruses that were found most frequently, SANTV and LEBV. These viruses are often sympatric, coexisting even in the same individual worm and individual cell. Sequence analysis of the collection of viral isolates did not detect genetic exchange between these two viruses but did detect reassortment of the two RNA molecules in SANTV. Finally, we developed a phenotypic assay that allowed us to detect an interaction between the host genotype and the SANTV variant.

(This article was submitted to an online preprint archive [17].)

## RESULTS

### The Mělník virus: a new noda-like virus infecting *Caenorhabditis briggsae*.

**FIG 1:**
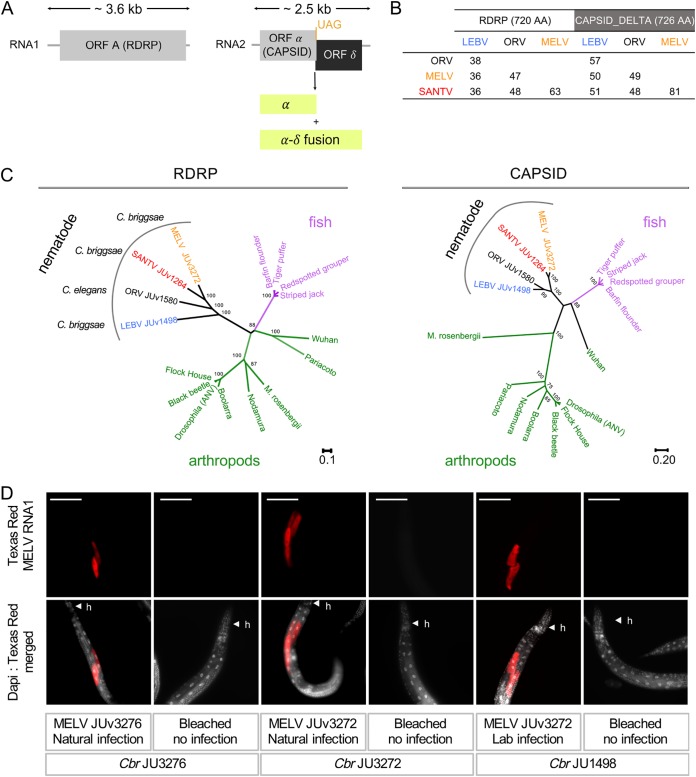
New *Caenorhabditis* noda-like virus: the Mělník virus. (A) Genome structure of the four noda-like *Caenorhabditis* viruses. (B) Pairwise percentage of amino acid (AA) identity between noda-like *Caenorhabditis* viruses. All positions containing gaps and missing data were eliminated. (C) Phylogenetic relationships between the RdRp and capsid amino acid sequences of classic nodaviruses and the four noda-like *Caenorhabditis* viruses. The delta ORF is not found in the classical nodaviruses. (D) Tissue tropism of the Mělník virus in C. briggsae (*Cbr*). The JU3272 and JU3276 C. briggsae isolates were naturally infected. The C. briggsae isolate JU1498 was experimentally infected with a filtrate of MELV JUv3272 from naturally infected JU3272. The three wild isolates JU3272, JU3276, and JU1498 were bleached (only embryos survive this treatment; see reference 25) and served as a control for the noninfected state. Mixed-stage populations were fixed after 5 days of culture at 23°C, and FISH staining was performed using one 21-nt probe (Texas Red) targeting the MELV RNA1 molecule. Nuclei were counterstained with 4′,6-diamidino-2-phenylindole (DAPI) (merged panels). h, head of C. briggsae animals. Red, MELV RNA1 probe with 200 ms exposition time; gray, DAPI staining. Scale bars represent 100 μm.

We sampled C. elegans and C. briggsae and searched for possible viral infections using host intestinal symptoms, *in situ* hybridization with probes corresponding to known viruses, and whole-RNA sequencing of infected cultures (see Materials and Methods). Based on intestinal symptoms (1) followed by RNA sequencing (see Materials and Methods), a novel C. briggsae virus, Mělník virus (MELV), was found in Mělník and Prague (Czech Republic). This virus causes the same intestinal symptoms as the other viruses (1), infects intestinal cells (Fig. 1D), and is not transmitted vertically, as shown by the loss of infection after submitting the culture to a bleach treatment. MELV is related to the three previously identified noda-like viruses. Partial genome sequences were obtained of 2,342 nucleotides (nt) for RNA1 and 2,257 nt for RNA2, which encompass a portion of the coding sequence for the RdRp and likely the entire capsid-delta proteins, respectively. We aligned the amino acid sequences of the RdRp and capsid-delta proteins of the four viruses (Fig. 1A and B for their pairwise similarity). Both the RdRp and capsid sequences of this new virus are most related to those of the Santeuil virus (Fig. 1B and C).

### Host specificity and geographical distribution of the four viruses.

In our surveys in France and the occasional sampling elsewhere in Europe, C. elegans was only found infected by ORV. *Caenorhabditis briggsae* was found infected by SANTV, LEBV, or MELV but never by ORV. We did not find intestinal infections by viruses in other *Caenorhabditis* species. This pattern of host specificity in natural populations matches the pattern of infection in the laboratory (1 and our results). For example, SANTV, LEBV, and MELV all infect C. briggsae JU1498 but do not infect the C. elegans ORV-sensitive strain JU1580. Conversely, the ORV variants JUv2572 and JUv1580 infect various C. elegans isolates (see below) but do not infect C. briggsae JU1264.

**FIG 2:**
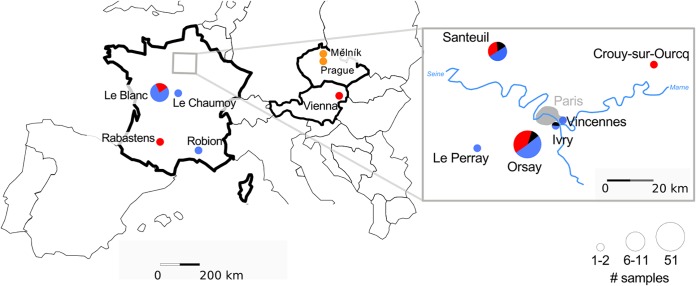
Geographical distribution of the sampled viruses. Pie charts represent the number of infected *Caenorhabditis*-containing samples collected per geographical location. The size of each pie mirrors the total number of positive samples per location, with three size classes: 1 to 2, 6 to 11, and 51 samples. The fractions of each pie represent the relative proportion for each virus recorded in this location. The viruses are color-coded: Orsay virus (ORV) in black, Santeuil virus (SANTV) in red, Le Blanc virus (LEBV) in blue, and Mělník (MELV) in orange. For a detailed list of strains, see Table S2.

Although C. elegans and C. briggsae were found at similar frequencies in France, viral infections of C. briggsae were more common than C. elegans infections (Fig. 2). Finding infected C. elegans was indeed rare, even in the highly resampled Orsay orchard. We only found it again in the Orsay orchard in 2014 and once in two other locations, Ivry and Santeuil (Fig. 2; see also Table S2 in the supplemental material).

**FIG 3:**
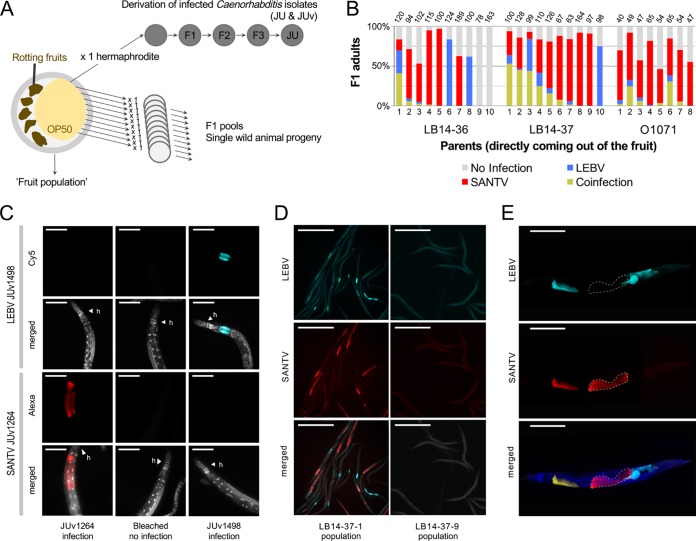
Coinfection of C. briggsae by Santeuil and Le Blanc viruses at the level of population, individual animals, and single cells. (A) Design of the assay. Rotten vegetal matter was collected from the wild, brought back to the laboratory, and plated on an E. coli OP50-seeded NGM plate. Single hermaphrodites of C. briggsae were isolated onto a new plate. C. briggsae wild isolates (JU) naturally infected with their noda-like viruses (JUv) were derived from the fruit population. In the survey for coinfection (shown in panel B), subsets of the C. briggsae wild populations coming out of the fruit (fruit population) were fixed in ethanol, and the presence of viruses was systematically monitored using *in situ* hybridization. (B) Natural coinfection in the progeny of wild animals (numbered on the *x* axis) isolated from rotten fruits. Three samples where SANTV and LEBV were present were chosen to assess the coexistence of the two viruses at the level of individual worms: a plum (LB14-36) and a pear (LB14-37) from the Le Blanc location and an apple (O1071) from the Orsay orchard. The F1 progeny (F1 pool) of 8 to 10 parents isolated from the parental populations were fixed in ethanol when they reached the adult stage. Many of the isolated adults harbored both viruses as assayed by their presence in their progeny, and some of their progeny also detectably harbored both viruses. Note that in all three cases the Santeuil virus appears predominant. The number of scored animals in each F1 pool is indicated on top of the bar. (C) Specificity of the ssDNA probes used to detect LEBV and SANTV. The JU1264 C. briggsae strain was experimentally infected with SANTV JUv1264 and with LEBV JUv1498 in the laboratory. Uninfected JU1264 C. briggsae animals were used as a control. Shown is fluorescence *in situ* hybridization staining of SANTV- and LEBV-infected animals using SANTV RNA1 (Cal Fluor red 610, 40×, 200 ms) and LEBV RNA1 (Quasar 670, 40×, 200 ms) Biosearch probes (see Table S1b). Nuclei were counterstained with DAPI (merged panels). Red, SANTV RNA1 probe; cyan, LEBV RNA1 probe; gray, DAPI. Scale bars represent 100 μm. (D) Coinfection at the level of the single C. briggsae LB14-37.1 F1 population. The uninfected C. briggsae F1 population LB14-37.9 was chosen as a control. Shown is fluorescence *in situ* hybridization staining of SANTV- and LEBV-infected animals using SANTV RNA1 (Cal Fluor red 610) and LEBV RNA1 (Quasar 670) probe sets (see Table S1b). Nuclei were counterstained with DAPI (merged panels). Red, SANTV RNA1 probe; cyan, LEBV RNA1 probe. Scale bars represent 500 μm. (E) Coinfections were recorded at the level of a single individual and a single cell both in naturally and in experimentally infected C. briggsae animals. Here, the illustration is of a C. briggsae adult hermaphrodite (strain JU1264) experimentally coinfected with JUv1264 and JUv1498 in the laboratory. Shown is fluorescence *in situ* hybridization staining of SANTV- and LEBV-infected animals using SANTV RNA1 (Cal Fluor red 610) and LEBV RNA1 (Quasar 670) probe sets (see Table S1b). Nuclei were counterstained with DAPI (merged panels). Dashed white lines delineate two intestinal cells infected with JUv1264 virus and not with JUv1498, exemplifying the probe specificity to each virus. Red, SANTV RNA1 probe; cyan, LEBV RNA1 probe; yellow, coinfected cells; blue, DAPI. Scale bars represent 100 μm.

C. briggsae is commonly found worldwide (14, 16), yet we rarely found signs of infection outside France and never outside Europe. Thus, our virus set is highly geographically biased toward Europe and particularly France. In France, we found SANTV and LEBV at similar frequencies in C. briggsae. The two viruses were found coexisting in the three most sampled locations: we found LEBV in the Santeuil wood, SANTV in Le Blanc, and both in the Orsay apple orchard (Fig. 2). Over the 2008–2014 collection period, we established from the Orsay orchard 20 C. briggsae isolates (strains or F1 progenies; see Materials and Methods and Fig. 3A) with SANTV only, 13 with LEBV only, and 10 with both. Including the other locations, we established 26 cultures with SANTV only, 27 with LEBV only, and 12 with both (Fig. 2 and Table S2).

### Cooccurrence of SANTV and LEBV in C. briggsae.

We first verified the specificity of each fluorescence *in situ* hybridization (FISH) probe set to detect either SANTV or LEBV RNA1 molecules (Fig. 3C). We then focused on sampling *Caenorhabditis* viruses in the Orsay orchard, where nematodes from a large set of 226 apples were systematically surveyed from 2010 to 2014 by *in situ* hybridization for the presence of SANTV and LEBV. In this set, 91/221 apples contained C. briggsae, and 43 of these C. briggsae populations were infected by a virus; out of these, 20/43 were infected by SANTV only and 13/43 by LEBV only, and 10/43 were coinfected by both viruses. Coinfection of C. briggsae fruit populations was also found in Le Blanc in 2014.

In order to assess the rate of C. briggsae coinfection at the scale of individual wild-caught animals, we then focused on three naturally coinfected populations (a pool of C. briggsae animals coming out of the rotting fruits LB14-37, LB14-36, and O1071) (Fig. 3). The coinfection rates in the progeny (F1) greatly differed among F0 individuals, ranging from no detectable infection to 50% coinfection (Fig. 3B; an example of coinfection signal in an F1 population is shown in Fig. 3D). In the highly infected population, LB14-37, 6 animals out of 10 had more than 10% of their progeny coinfected. In the moderately infected populations, LB14-36 and O1071, only 1/10 and 2/8 animals had more than 10% of their progeny coinfected, suggesting that the coinfection of a fruit population can be lost relatively quickly when establishing an infected *Caenorhabditis* isolate (JU and its virus, JUv), as shown in Fig. 3A.

We next asked whether the two viruses could be found in the same individual nematode or in the same intestinal cell. Our results show that SANTV and LEBV could be found infecting the same cell in naturally infected C. briggsae animals (Fig. 3B to D) as well as in C. briggsae isolates simultaneously infected by both viruses under laboratory conditions (Fig. 3E).

### Genetic diversity, phylogeny, and reassortments.

We obtained, by Sanger sequencing, nearly the entire RNA1 and RNA2 segments for a large set of the collected viruses (listed in Table S1a). We did not find any evidence of genetic exchange between the different *Caenorhabditis* noda-like viruses using RDP4 (18). Indeed, SANTV always contained both RNA1 and RNA2 molecules that were closely related to our original SANTV strain, JUv1264 (1), and the situation was similar for LEBV (2). Moreover, we did not find any evidence of genetic exchange between the different *Caenorhabditis* noda-like viruses when we screened natural populations by FISH staining of SANTV- and LEBV-infected animals using RNA1 and RNA2 probes shown in Table S1b.

**FIG 4:**
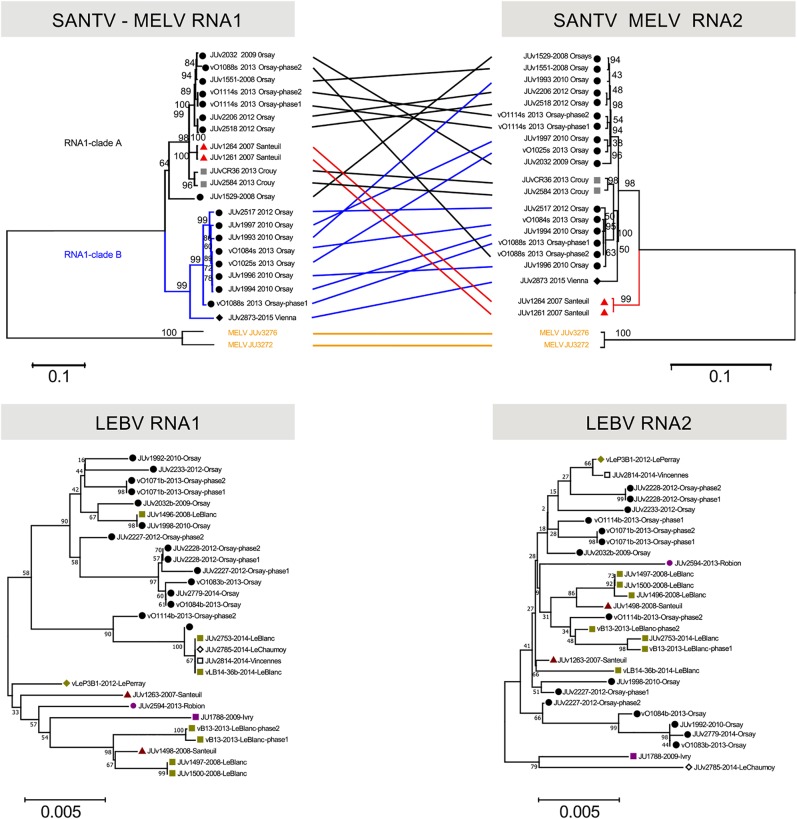
Phylogenetic relationships of the SANTV and LEBV variants. (Top) Tanglegram of SANTV RNA1 and RNA2 trees rooted with MELV. (Bottom) Trees for LEBV RNA1 and RNA2 (here, no tanglegram was built, because the trees are less well supported due to lower polymorphism). The phylogenetic relationships were inferred using the neighbor-joining method (see Materials and Methods). All positions containing gaps and missing data were eliminated. The percentage of replicate trees in which the associated taxa clustered together in the bootstrap test (10,000 replicates) are shown next to the branches. The evolutionary distances were computed using the maximum composite likelihood method. The tree is drawn to scale with the number of base substitutions per site. The locations are coded by a colored shape next to the name of the variant. For SANTV, the tanglegram was drawn manually. For SANTV, the branches leading to variants in clade B of RNA1 are labeled in blue and those in the clade A are in black, with the exception of JUv1264 and JUv1261, in red. The variants clustering in the RNA1 tree do not cluster in the RNA2 tree, indicating reassortment between RNA1 and RNA2. The MELV variants are labeled in yellow. All positions with less than 90% site coverage were eliminated. The total of positions used to build trees were of 2,877 nt for SANTV RNA1, 2,251 nt for SANTV RNA2, 2,976 nt for LEBV RNA1, and 2,523 nt for LEBV RNA2.

Within each viral species, we found extensive genetic diversity among the strains. The highest genetic diversity was found for the SANTV RNA1 molecule, with a total of 21% of nucleotides (11.9% of amino acids) showing a polymorphism in the whole sequenced set. Mean pairwise diversity (± standard errors [SE]) for the entire sample was 0.086 ± 0.019 substitutions per site at the nucleotide level and 0.035 ± 0.005 at the amino acid level. The pattern of SANTV RNA1 diversity includes a deep split between two lineages, which we call A and B (Fig. 4). The estimate of evolutionary divergence over sequence pairs between RNA1 of lineages A and B was 0.099 ± 0.037. The RNA1 lineages A and B had similar mean diversity of 0.0024 ± 0.002 and 0.0024 ± 0.007 substitutions per site at the nucleotide level and of 0.012 ± 0.002 and 0.014 ± 0.002 at the amino acid level, respectively. Lineage A was found in Orsay in 2008, 2009, 2010, 2012, and 2013 and in several other locations, while lineage B was found in Orsay in 2010 and 2013 and Vienna in 2017.

The SANTV RNA2 molecule displayed a lower level of variation than RNA1: mean diversity for the entire sample was 0.027 ± 0.002 substitutions per site at the nucleotide level and 0.011 ± 0.002 at the amino acid level. Interestingly, the phylogeny of RNA2 was not congruent with that of RNA1, suggesting reassortments (Fig. 4). Using RDP4 (18), we indeed detected five reassortment events between the RNA1 and RNA2 molecules (Table S3). No significant intramolecular recombination was detected in our panel of viral variants. We did find populations from a single fruit that were infected by two variants (e.g., vO1088s in Fig. 4). Thus, it is likely that SANTV variants reassort while coinfecting the same individual nematode.

For LEBV, despite a similar sampling structure and geographic diversity, we found a much lower level of genetic diversity than that for the Santeuil virus on both RNA molecules. The RNA1 molecule displays 0.013 ± 0.001 substitutions per site at the nucleotide level and 0.007 ± 0.001 at the amino acid level, while RNA2 harbors 0.011 ± 0.001 substitutions per site at the nucleotide level and 0.007 ± 0.001 at the amino acid level. The topology of the phylogenetic relationships between LEBV variants for RNA1 and RNA2 was poorly supported, possibly because of the lower number of informative sites (Fig. 4). Using RDP4 (18), we detected one event of reassortment between RNA1 and RNA2 molecules (Table S3).

**FIG 5:**
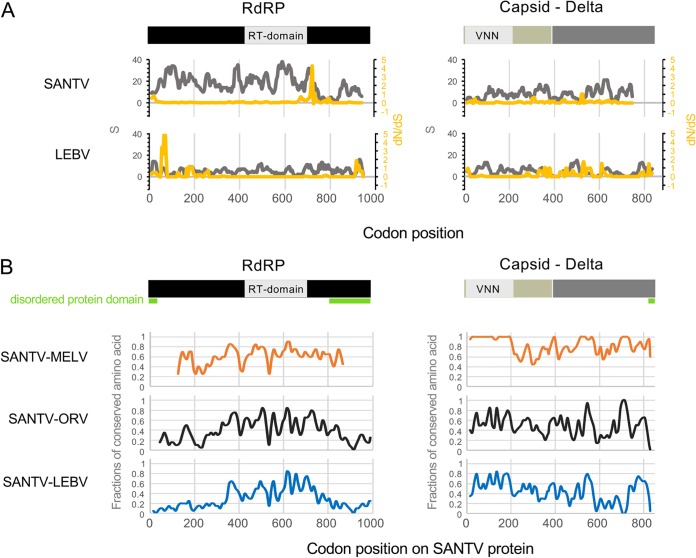
Pattern of molecular diversity along the open reading frames. (A) The total number of mutations (*S*) are indicated in gray, and the ratios of nonsynonymous to synonymous substitutions (*dN*/*dS*) in yellow, where *dS* is the number of synonymous substitutions per site (*s*/*S*) and *dN* is the number of nonsynonymous substitutions per site (*n*/*N*). Values were calculated for the RNA1 (RdRp) and RNA2 (capsid-delta fusion protein) molecules of SANTV and LEBV in a sliding window, with a window of 20 codons and a step of 5 codons. (B) The fractions of amino acids conserved between SANTV and the other noda-like viruses (MELV, ORV, and LEBV) along the RNA1 and RNA2 molecules were estimated in a sliding window (20 amino acids to 10 amino acids). RdRp is in black, including the RT (reverse transcriptase) domain. Capsid is in beige, including the VNN (viral nervous necrosis) domain that belongs to the viral coat superfamily (S domain), and delta is in dark gray. Disordered protein domains as predicted by the PrDOS protein disorder prediction server (http://prdos.hgc.jp) are underlined in green.

The pattern in the number of polymorphic sites (*S*) and the ratio of nonsynonymous to synonymous substitutions (*dN*/*dS*) along the RNA1 and RNA2 molecules are shown for the LEBV and SANTV variants in Fig. 5A. Perhaps most striking is the low level of synonymous polymorphism compared to nonsynonymous polymorphisms around codon 725 of SANTV RNA1 and 70 of LEBV RNA1. However, the [*dN-dS*] statistical test implemented in the HyPhy package (19) did not detect any positive selection in our data set. The Z-test (codon-based tests of positive and purifying selection for analysis averaging over all sequence pairs) rejected the null hypothesis of strict neutrality in favor of purifying selection for RNA1 and RNA2 of both LEBV and SANTV.

Finally, the patterns of pairwise amino acid conservation between SANTV and MELV, ORV, or LEBV along the RNA1 and RNA2 molecules are shown in Fig. 5B. No amino acid region is specifically conserved between the three C. briggsae viruses (SANTV, MELV, and LEBV) that is not also shared with the C. elegans virus (ORV). Remarkably, the N-terminal capsid domain is highly conserved between the two closely related viruses MELV and SANTV.

### Phenotypic assays of viral variant infections in different hosts.

We observed that some host-virus combinations caused more damage to the host than others, as revealed by intestinal symptoms and slow growth. We previously reported that C. elegans wild isolates display a wide range of sensitivity to the original ORV strain, JUv1580 (3), and we will report elsewhere on differential sensitivity observed in C. briggsae (see also reference 1). Here, we aimed to test the effect of genetic diversity within viral species in different hosts. To compare viral variants, we were limited by the fact that the reverse transcription-quantitative PCR (RT-qPCR) titer of our filtered viral preparations may not reflect the amount of infectious viral particles. To circumvent this titration problem, we used several protocols that aimed at measuring viral production by a given host independently of the original viral titer.

**FIG 6:**
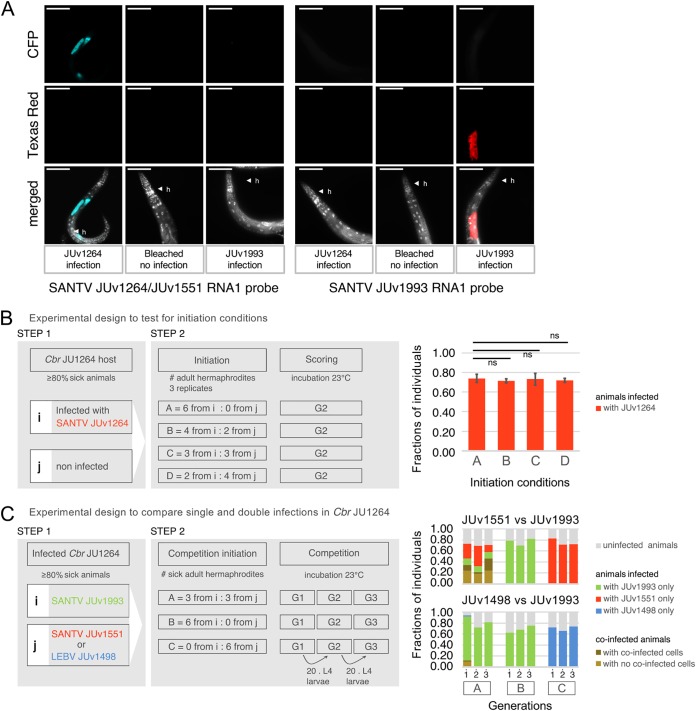
Test of the initial conditions for pairwise infection assays. (A) Specificity of the ssDNA probes used to detect SANTV variants. The JU1264 C. briggsae strain was experimentally infected with SANTV JUv1264 and with SANTV JUv1993 in the laboratory. Uninfected JU1264 C. briggsae animals were used as a control. Shown is fluorescence *in situ* hybridization staining of SANTV JUv1264- and JUv1993-infected animals using JUv1264/JU1551 RNA1 (CFP/ATTO425, 40×, 200 ms) and JUv1993 RNA1 (Texas Red, 40×, 200 ms) 22-nt-long probes (see Table S1c). Nuclei were counterstained with DAPI (merged panels). Red, SANTV JUv1264; cyan, SANTV JUv1264; gray, DAPI. h, head of the animal. Scale bars represent 100 μm. (B, left) Design of the assay to test the effect of the number of sick animals (viral titer) to initiate the infection in C. elegans JU1264 with SANTV JUv1264. Infections were performed in triplicates. Results are shown in the right panel. Bars represent the standard deviations among replicates. ns, no significant differences found between the final number of sick animals recorded from the initial viral titers, using analysis of variance and multiple comparisons of means with Tukey contrasts. (C, left) Design of the assay to compare virus infectivity in single infection or in competition. Infections were performed in triplicates. (Right) Result of the above-described experiment in C. briggsae JU1264, expressed as a fraction of infected individuals across three generations. SANTV JUv1551 and JUv1993 were maintained at similar frequencies (top), whereas LEBV JUv1498 tended to lose to SANTV JUv1993 (bottom). Numbers 1, 2, and 3 designate the passage generation of the population. (Co-)infection levels of SANTV versus LEBV-infected animals were scored using FISH staining with SANTV RNA1 (Cal Fluor red 610, 40×, 200 ms) and LEBV RNA1 (Quasar 670, 40×, 200 ms) Biosearch probes (see Table S1b). (Co-)infection levels of JUv1551- versus JUv1993-infected animals were scored using FISH staining with JUv1264/JU1551 RNA1 (CFP/ATTO425, 40×, 200 ms) and JUv1993 RNA1 (Texas Red, 40×, 200 ms) 22-nt-long probes (see Table S1c), such as those described for panel A.

First, in the simplest protocol, we established single infections and monitored the proportion of infected animals after 2 to 3 host generations so that the final host infection rate is a function of the host-pathogen interactions and not of the initial viral titer (i.e., number of sick animals used as the input) (1, 3) (Fig. 6B; see also Fig. 8). In single infections in C. briggsae JU1264, the SANTV and LEBV variants reached an infection rate of 70 to 80% of the animals, a percentage that is indeed independent of the initial titer over the tested range (Fig. 6B) and independent of the virus variant among SANTV JUv1264, JUv1551, JUv1993, and LEBV JUv1498 (Fig. 6B and C).

**FIG 7:**
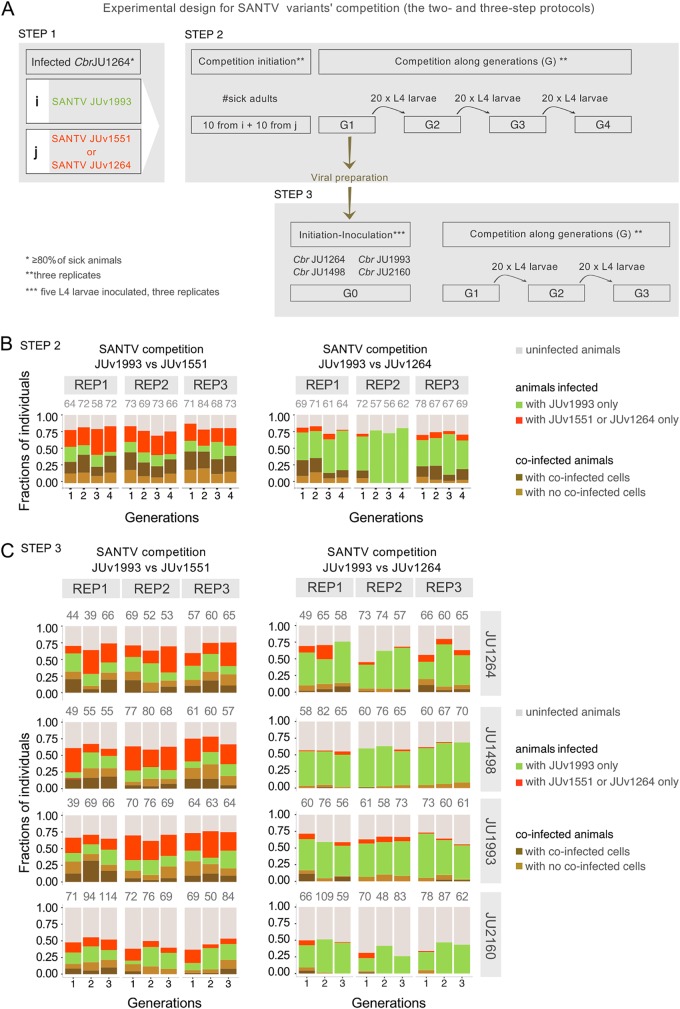
Outcome of competition between two SANTV variants depends on both the Santeuil virus genotypes and the host genotype. (A) Design of the assay for the pairwise competition between the JUv1993 SANTV variant and each of the two SANTV variants, JUv1264 (variant of reference) and JUv1551. JUv1993 and JUv1551 were chosen because they possess highly divergent RNA1 and similar RNA2 molecules (Fig. 4). The results are shown in panel B. The viral preparation (in dark yellow) was then used to inoculate five L4 larvae of four different C. briggsae hosts, JU1264, JU1498, JU1993, and JU2160. The results are shown in panel C. (B) Result of the above-described experiment performed in triplicate in C. briggsae JU1264, expressed as a fraction of infected individuals across four generations. The Santeuil virus variants JUv1551 and JUv1993 were maintained at similar frequencies (left), whereas JUv1264 tends to lose to JUv1993 (right). (C) The tested SANTV genotypes are indicated above the graph, and the C. briggsae host genotype is on the right. In panels B and C, 0, 1, 2, and 3 designate the passage generation of the population, with generation 1 being the progeny of the originally infected animals. REP stands for replicate. All experiments were performed in parallel. (Co-)infection levels were scored using FISH staining of SANTV-infected cells/animals using single-oligonucleotide probes targeting JUv1264/JUv1551 RNA1 (ATTO425) and JUv1993 RNA1 (Texas Red) (see Table S1c). The total numbers of animals scored per replicate are given above the histograms.

Second, we established coinfections where two virus variants competed in the same host (Fig. 6C and 7B). We started the coinfections using preinfected animals from two singly infected stable populations (step 1), where ca. 80% of the animals were infected after 7 days (2 to 3 generations) (see Materials and Methods) (Fig. 6C and 7A). We then mixed together sick adult animals of the singly infected populations (step 2) and let them reproduce for 3 days. We then passed 20 L4 larvae from the population every 3 days for three generations. In each generation, ca. 100 animals were then fixed for *in situ* hybridization. To be able to monitor two SANTV variants, we developed probes specific for two divergent SANTV RNA1 from the two clades shown in Fig. 4A (Fig. 6A and Table S1C). In these coinfections, each virus variant infected fewer animals than single infections. Two examples of balanced and unbalanced outcomes are shown in Fig. 6C and 7B.

With this assay, we observed reproducible differences between SANTV genotypes. JUv1551 and JUv1993 RNA1 were both maintained quite stably over the four generations, and both infected similar proportions of infected animals and numbers of cells. In the JUv1993 versus JUv1264 competition, more animals were infected by JUv1993 RNA1 than by JUv1264 RNA1; the latter even disappeared by the second generation in one replicate (Fig. 7B). We conclude that JUv1264 has a lower ability to infect the C. briggsae JU1264 host in this two-step protocol when faced with JUv1993. The effect may be due in part to a lower single infection rate by JUv1264 than by JUv1993 in step 1 and in part to a lower competitive ability of JUv1264 in step 2.

Third, we sought to compare the capabilities of these viruses in several C. briggsae host strains by starting a new infection from the mixed viral preparation in the first generation of coinfection from the previous experiment (Fig. 7C, red). We infected in parallel C. briggsae strains JU1264 (originally infected by SANTV), JU1498 (by LEBV), JU1993 (by SANTV), and JU2160 (tropical isolate from Zanzibar). Consistent with the above-described results, the Santeuil virus genotypes JUv1551 and JUv1993 were comaintained in C. briggsae JU1264 in similar proportions of infected animals across the three generations, while more animals were infected by JUv1993 than by JUv1264 (Fig. 7C). The same result applied when the JU1993 host was infected. Interestingly, the viral genotype JUv1993 appeared to outcompete JUv1264 better in the host strain JU1498 than in JU1264 (generalized linear model using a logistic regression on the number of cells respectively infected with JUv1993 and JUv1264 and a quasibinomial model with logit link; *P* = 0.006) and even better in the host strain JU2160, where the JUv1264 variant was lost in all three replicates by the third generation (glm using a logistic regression on the number of cells infected with JUv1993 and JUv1264 and a quasibinomial model with logit link; *P* = 0.024).

In summary, these experiments indicate an interaction between host genotype and SANTV genotype, such that the result of the competition depends on the host strain.

### A new ORV variant with different infection characteristics.

**FIG 8:**
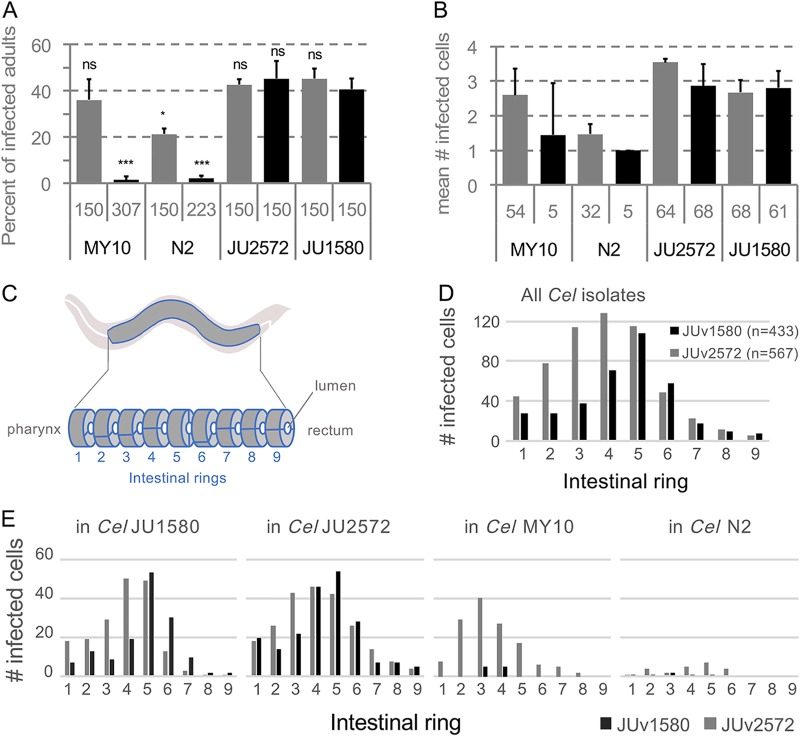
Comparison of ORV variant infections in different C. elegans wild isolates. N2, JU1580, MY10, and JU2572 C. elegans wild isolates were infected with the ORV variants JUv2572 and JUv1580. For each C. elegans isolate, five L4 larvae were infected with 50 μl of the ORV viral filtrate (either JUv1580 or JUv2572), and cultures were propagated for two/three generations at 20°C. Infections were performed in triplicates. ORV infections were monitored on F2 adult hermaphrodites by FISH using a Biosearch probe (Cal Fluor red 610) targeting the ORV RNA2 molecule. (A) Proportion of infected F2/F3 adult hermaphrodites. The total number of animals scored are given below the histograms. Bars represent the standard deviations among replicates. *P* < 0.001 (***) and *P* < 0.05 (*) in a glm taking the viral variant knowing the host genotype into account, with each virus effect being compared to JUv1580 on the host C. elegans JU1580. (B) Number of infected cells per infected F2/F3 adult hermaphrodite. The total number of infected animals are given below the histograms. (C) At the adult stage, the intestine of Caenorhabditis elegans is composed of 9 rings of intestinal cells (2 or 4), ring 1 being close to the pharynx and ring 9 to the rectum. (D and E) Distribution of infected cells along C. elegans (*Cel*) intestine considering all the C. elegans (D) isolates or each of them separately (E).

Concerning the ORV, we noticed that the Ivry JU2572 C. elegans isolate infected with ORV JUv2572 harbored stronger intestinal symptoms and slower growth than most ORV infections. Using the single infection protocol, the viral preparation from the JU2572 isolate was able to infect the strains N2 and MY10, which are more resistant to infection by JUv1580 (3) (Fig. 8A and B). Note that we never obtained infection of MY10 with any JUv1580 preparation, while N2 was variably infected (1, 3). Interestingly, JUv2572 tended to infect its host intestine more anteriorly than JUv1580, and this was independent of the C. elegans isolate infected (Fig. 8C to E). Thus, this new ORV isolate may constitute a useful resource for C. elegans viral infections.

## DISCUSSION

To date, four *Caenorhabditis* noda-like viruses have been discovered. The Santeuil, Orsay, and Le Blanc viruses and the new Mělník virus share the same tissue tropism toward intestinal cells and are horizontally transmitted. The capsid-delta protein is more conserved among them than the RdRp protein. MELV is particularly close to SANTV for open reading frames located on RNA2, especially the first 207 amino acids of the capsid protein. However, their nucleotide sequences are much less conserved, and we did not detect any putative recombination event using RDP4. This suggests that the evolution of this part of the capsid is more constrained. While ORV infects C. elegans and the other three viruses infect C. briggsae, the ORV and LEBV capsid-delta proteins share more similarities with each other than with SANTV and MELV. The amino acid conservation and phylogenetic positions of the four viruses suggest that ORV is derived from LEBV while specializing on the host, C. elegans. Further comparison between LEBV and ORV, including reconstitution of infection through transgenes, as described in reference 8, therefore could provide a better understanding of the host switch from C. briggsae to C. elegans.

Although C. elegans and C. briggsae are found at similar frequencies in France (20, 21), we mostly recorded viral infections of C. briggsae. The number of viral infections recorded for C. elegans was low even if, under laboratory conditions, many European C. elegans wild isolates are sensitive to the Orsay virus (3). In controlled infections, we commonly observed higher infection rates of C. briggsae by SANTV or LEBV than of C. elegans by ORV (e.g., 60 to 80% versus 30 to 50%, respectively, of the animals infected at 7 days after infection; examples are in Fig. 6 and 8). The differences in infectivity between SANTV and LEBV on one hand and ORV on the other hand could explain in part the lower probability to detect infected C. elegans than infected C. briggsae. Note that here we found that preparations of the ORV JUv2572 from JU2572 are able to infect strains such as C. elegans N2 and MY10 better than JUv1580 preparations (Fig. 8). This may alleviate some previous issues raised with the use of the latter (22). The more anterior tropism within the intestine of adult hosts is an interesting and unexplained feature.

The C. briggsae species is distributed all over the world (16), yet we rarely found signs of infection outside France and never outside Europe. Whether this geographical bias in virus sampling reflects the geographical distribution of the sensitivity of C. briggsae to the noda-like viruses is still to be clarified. On another note, the time between the collection in the field and the processing of the sample (rotting vegetal matter) in the laboratory could impact the survival of the *Caenorhabditis* animals, especially for the infected animals, and therefore lower the probability of recovering infected animals. Variation in host sensitivity (3) could be another factor.

The Santeuil and Le Blanc viruses are often sympatric in France, coexisting even in the same individual worm and individual cell. Thus, they are in principle susceptible to exchanging genetic material. Reassortment, by shuffling viral RNA molecules between various species, likely plays an important role in viral evolution (23, 24). The reassortment of RNA molecules (horizontal gene transfer) can occur between highly divergent viruses. For instance, the mosinovirus (MoNV) originated from the reassortment between a virus closely related to the Pariacoto virus (*Nodaviridae*) (MoNV RdRp shares 43% amino acid identity with Pariacoto virus) and a virus closely related to Lake Sinai virus 2 (MoNV capsid shares 16% amino acid with the LSV 2) (23). In our study, sequence analysis did not detect genetic exchange between SANTV and LEBV but did detect several reassortment events between SANTV variants.

Here, we also designed assays that allow us to study the interaction between the different viral variants and hosts. This allowed us to determine that the outcome of the competition between SANTV variants depended on the C. briggsae host.

*Caenorhabditis* nematodes and their noda-like viruses provide an exciting model to investigate coevolution dynamics between an animal host and its natural viruses. First, C. briggsae and C. elegans are model organisms with a short life cycle, which eases multigenerational experiments and quantitative genetic analysis. Second, the natural combinations of one *Caenorhabditis* isolate with its natural viral variant can easily be isolated, maintained under laboratory conditions, kept frozen, and revived. Third, natural virus-nematode combinations can be dissociated by storing the viral isolate as a filtrate and by bleaching the *Caenorhabditis* isolate to remove viral infections (as in reference 25). Fourth, the infection can be reconstituted by transgenesis (8), allowing researchers to test the effect of precise sequence changes. Fifth, we developed here a phenotypic assay to test the competitive ability of two viruses in different host genotypes. This leads the way to further studies of phenotypically significant evolutionary change in both host and virus.

## MATERIALS AND METHODS

### Viral strain nomenclature.

The names of the viruses are abbreviated as ORV for the Orsay virus, SANTV for the Santeuil virus, LEBV for the Le Blanc virus, and MELV for the Mělník virus. The different viral strains were designated using the code JUv0000, where the viral strain comes from the nematode strain JU0000. In cases of coinfection of the nematode strain JU0000, we used JUv0000b and JUv0000s to distinguish between the LEBV and SANTV strains, respectively. We found the viruses in different locations, including the original locations after which they are named. To avoid confusion between the viruses and the locations, we always specify SANTV when it is the virus, whereas Santeuil alone designates the location. Finally, viral samples obtained from a population of *Caenorhabditis* coming from one rotting fruit were coded vX0000s and/or vX0000b.

### Sampling of nematodes and associated viruses.

C. elegans and C. briggsae natural populations were collected as described previously (15, 20). We collected them worldwide but with a strong geographical bias toward France. In this area, C. elegans and C. briggsae are found to be the predominant *Caenorhabditis* species (14–16, 20, 21). The presence of viruses was deduced from intestinal symptoms (1), especially when observed in the absence of other visible pathogens, such as microsporidia (26) and bacteria (27), and confirmed using *in situ* hybridization (see below).

In the Orsay orchard 2013 survey, the presence of viruses was systematically monitored in C. briggsae using *in situ* hybridization (see below) performed directly on a subset of worms coming out of the rotten fruits. The fixed worm population was split in two; half was hybridized with SANTV RNA1 plus RNA2 probes and the other half with LEBV RNA1 plus RNA2 probes (see Table S1b in the supplemental material). The cultures were propagated on agar plates seeded with Escherichia coli OP50 and frozen under standard conditions for C. elegans (25).

### Detection and *de novo* sequencing of a new virus, the Mělník virus.

In order to detect unknown viruses of C. briggsae and C. elegans, total RNAs were extracted from mixed-stage populations of C. elegans and C. briggsae wild isolates. These isolates were pooled, and sequencing libraries were constructed and sequenced on a 2- by 250-bp Illumina Miseq platform. They then were screened for viral sequences using VirusSeeker (28). Sequences related to SANTV were detected in C. briggsae strains JU3272 and JU3276, here referred to as Mělník virus. Using RT-PCR and 3× Sanger sequencing, partial genomes of the MELV RNA1 and RNA2 sequences were obtained (JUv3272, GenBank accession numbers MK774657 and MK774659; JUv3276, MK774658 and MK774660).

### Detection of viruses by fluorescent *in situ* hybridization (FISH).

Each virus was detected by *in situ* hybridization using fluorescently labeled oligonucleotides specific for each virus, as described in reference 7. The list of probes is found in Table S1. A single probe is sufficient to detect infection. To enhance sensitivity of the wild isolate screens, especially in the case of sequence variation, multiple (48 or 32) oligonucleotides along the viral RNA genome were used.

Fluorescence microscopy was performed using an upright Zeiss AxioImager M1 equipped with 10× (0.3 numerical aperture), 40× (1.3 numerical aperture), and 63× (1.25 numerical aperture) objectives. Images were acquired using a Pixis 1024B camera (Princeton instruments) and MetaVue imaging software. Image panels were assembled in ImageJ (29) and Inkscape (0.91 r13725; www.inkscape.org) software. Animals were considered infected when, for an exposure below or equal to 1,000 ms with the 40× objective, at least one intestinal cell was distinctly labeled at higher levels than background staining for that animal (i.e., the signal recorded in the germ line and pharynx of the same animal).

To screen for the natural C. elegans infections with ORV, we used the custom Stellaris (Biosearch Technologies) probes labeled with Quasar 670 dyes for the ORV RNA1 molecule and with Cal Fluor red 610 dyes for the ORV RNA2 molecule described in reference 7.

To screen for the natural C. briggsae infections with SANTV and LEBV, we used custom Stellaris (Biosearch Technologies) probes labeled with Quasar 670 dyes for the SANTV RNA1 and LEBV RNA2 molecules and with Cal Fluor red 610 dyes for the SANTV RNA2 and LEBV RNA1 molecules. FISH was performed as described in reference 30, except that the hybridization solution contained 20% formamide on each fixed sample with a 7-h hybridization at 30°C with the Biosearch probes, targeting either the SANTV and LEBV RNA1 molecules or the SANTV and LEBV RNA2 molecules.

For the MELV, we developed a custom Eurofins 21-nt-long probe labeled with Texas Red dye for the MELV RNA1 molecule (Table S1c).

For viral variant competition experiments, we developed custom Eurofins 22-nt-long probes specific for two divergent SANTV RNA1 variants, labeled with CFP-ATTO425 for JUv1993s and with Texas Red for JUv1264s and JUv1551s. The probe sequences are provided in Table S1c.

### Test for DNA probe specificity.

In order to test the specificity of the DNA probes used to detect LEBV and SANTV, JU1264 C. briggsae animals were experimentally infected either with SANTV JUv1264 or with LEBV JUv1498 or left uninfected. The SANTV-infected, LEBV-infected, and noninfected animals were fixed, and we performed fluorescence *in situ* hybridization staining using SANTV RNA1 (Cal Fluor red 610) and LEBV RNA1 (Quasar 670) Biosearch probes (Table S1b).

In order to test the ability of our 22-nt-long single-stranded DNA (ssDNA) probes to distinguish between SANTV JUv1993 RNA1 and SANTV JUv1264 or JUv1551 RNA1 molecules, we infected the JU1264 C. briggsae strain with either SANTV JUv1264 or with SANTV JUv1993 or left the animals uninfected. The SANTV JUv1264- or JUv1993-infected and the noninfected animals were fixed, and we performed fluorescence *in situ* hybridization staining using the JUv1993 RNA1 (Texas Red) and JUv1264/JUv1551 RNA1 (CFP) probes (Table S1c).

### Rate of C. briggsae coinfection with the Santeuil and Le Blanc viruses at the fruit scale.

To assess the rate of C. briggsae coinfection with SANTV and LEBV at the scale of the fruit, we focused on two samples from the Le Blanc location, LB14-36 (plum) and LB14-37 (pear), and one from the Orsay orchard, apple O1071. Wild hermaphrodite L4 larvae were singled less than 4 h after the rotten vegetal matter was placed onto E. coli OP50-seeded nematode growth medium (NGM) plates maintained at 23°C. After 3 days, F1 progenies were fixed with ethanol. To monitor the presence of LEBV and SANTV, FISH was performed as described in reference 30, except that the hybridization solution contained 20% formamide, with a 7-h hybridization at 30°C of the custom Stellaris (Biosearch Technologies) probes labeled with Quasar 670 dyes for the SANTV RNA1 molecule and with Cal Fluor red 610 dyes for the LEBV RNA1 molecule (Table S1b).

### Sequencing of viral variant genomes.

RNAs of a subset of infected *Caenorhabditis* wild isolates were extracted using TRIzol-chloroform. Viral genomes were first reverse transcribed from the 3′ end, and from the middle of the segment when needed, using viral species-specific primers and Superscript III reverse transcriptase (Invitrogen) by following the manufacturer’s instructions. Reverse transcription was followed by PCR amplifications using the Q5 high-fidelity DNA polymerase (New England Biolabs) with primers amplifying two overlapping fragments per RNA segment. The PCR products were Sanger sequenced. To minimize sequence errors due to PCR, we performed two independent reverse transcription-PCR experiments but did not find any mismatches between replicates, and we were able to validate heterozygous single-nucleotide polymorphisms. All of the primers used in this study are listed in Table S1a. Sequences are available under the following GenBank accessions numbers: MN130962 to MN130984 (LEBV-RNA1), MN130985 to MN131007 (LEBV-RNA2), MN131008 to MN131027 (SANTV-RNA1), and MN131028 to MN131047 (SANTV-RNA2).

### Analyses of viral genetic diversity.

**(i) Alignments and phylogenetic analyses.** Nucleotide sequences were aligned using MUSCLE (31) with default parameters implemented in MEGA7 (32). When necessary, heterozygous sequences were phased using fastphase implemented in DNAsP v5 (33). The sequence relationships were inferred in MEGA7 (32) using the maximum likelihood method based on the Jones-Taylor-Thornton (JTT) matrix-based model (34) and tested using 10,000 bootstraps. The initial tree(s) for the heuristic search was obtained automatically by applying neighbor-joining algorithms to a matrix of pairwise distances estimated using a JTT model and then selecting the topology with the superior log likelihood value. Time trees of SANTV and MELV phylogenetic relationships were inferred using the Reltime method (35) and estimates of branch lengths inferred using the neighbor-joining method (36).

**(ii) Detection of reassortments.** To detect events of RNA molecule reassortment between variants of the same viral species, we concatenated, for each variant, their RNA1 and RNA2 nucleotide sequences. We detected putative reassortment and recombination events using the RDP, GENECONV, Bootscan, Maxchi, Chimera, SiSscan, and 3Seq methods implemented in RDP4 (18) software. For all these methods, the following parameters were used: neighbor-joining tree was built using 1,000 bootstraps, and events were considered significant for a *P* value of less than 0.001.

**(iii) Analysis of polymorphisms along the ORFs within the set of LEBV and SANTV variants.** Mean evolutionary diversities for the entire populations and mean interpopulational evolutionary diversities were estimated using MEGA7 (32) by following Nei and Kumar calculations (37). We calculated the number of base substitutions per site and the number of amino acid or nucleotide substitutions per site from mean diversity calculations for the entire population and, when specified, from mean interpopulational diversity. Standard error estimates were obtained by a bootstrap procedure (1,000 replicates). Analyses were conducted using the maximum composite likelihood model (38) for nucleotides and using the JTT matrix-based model (34) for amino acids. All positions with less than 90% site coverage were eliminated, i.e., fewer than 10% alignment gaps, missing data, and ambiguous bases were allowed at any position.

The total number of mutations (*S*) and the ratios of nonsynonymous to synonymous substitutions (*dN*/*dS*), where *dS* is the number of synonymous substitutions per site (*s*/*S*) and *dN* is the number of nonsynonymous substitutions per site (*n*/*N*), were calculated for the RNA1 (RdRp) and RNA2 (capsid-delta fusion protein) molecules of SANTV and LEBV in a sliding window, with a window of 20 codons and a step of 5 codons. In order to describe the distribution of the total, synonymous, and nonsynonymous polymorphisms, we used the maximum likelihood analysis of natural selection codon by codon using the HyPhy software package (19) implemented in MEGA5 (39). For each codon, estimates of the numbers of inferred synonymous (*s*) and nonsynonymous (*n*) substitutions and the numbers of sites that are estimated to be synonymous (*S*) and nonsynymous (*N*) were produced using the joint maximum likelihood reconstructions of ancestral states under a Muse-Gaut model (40) of codon substitution and the Felsenstein model (41) of nucleotide substitution. For estimating maximum likelihood values, a tree topology was automatically computed. To detect codons that have undergone positive selection, we used the [*dN-dS*] statistical test implemented in the HyPhy software package (19). The *dN*/*dS* ratio values were then calculated for a sliding window with a window of 20 bp and a step of 1 bp.

The codon-based tests of positive and purifying selection averaging over all sequence pairs were conducted in MEGA7 (32), providing the probabilities of rejecting the null hypothesis of strict neutrality (*dN* = *dS*) in favor of an alternative hypothesis (*dN* > *dS* = 1.00 or *dN* < *dS* = 1.00). The variance of the difference was computed using the bootstrap method (10,000 replicates). Analyses were conducted using the Nei-Gojobori method (42). All ambiguous positions were removed for each sequence pair.

### Viral preparations.

Naturally infected C. briggsae wild isolates were thawed and propagated for 7 days onto OP50-seeded NGM plates at 23°C. Viral preparations were made from these cultures as described in reference 3 for the following viruses: SANTV (JUv1264, JUv1993, and JUv1551), LEBV (JUv1498), ORV (JUv1580 and JUv2572), and MELV (JUv3272).

### Phenotypic assays of viral variant infections in different hosts.

**(i) Infections of four C. elegans strains with two ORV variants.** We established C. elegans infections with two ORV variants. Five L4 larvae were infected with 50 μl of viral preparation. Infected C. elegans populations were propagated onto OP50-seeded NGM plates at 20°C for 2 to 3 host generations so that the final titer is a function of the host-pathogen interaction rather than of the initial titer (1, 3). The original host strain, C. elegans JU1580, and three other C. elegans strains, N2, JU2572, and MY10, were infected with the ORV JUv2572 strain from Ivry and with the original ORV JUv1580 isolate from the Orsay orchard (Fig. 8). F2/F3 populations were harvested and fixed for *in situ* hybridization using custom Stellaris probes targeting ORV RNA2 molecules (Table S1b). The number of infected cells was counted manually.

**(ii) Competition between SANTV and LEBV in different C. briggsae hosts.** We first performed a two-step infection experiment to test the effect of the initial viral titer (i.e., number of sick animals) on infection rate after 2 to 3 host generations. We infected C. briggsae JU1264 animals with the JUv1264 viral preparation in three replicates. We visually assessed symptoms on 50 adult hermaphrodites and checked that after 7 days more than 80% of the nematodes were infected. We then mixed *i* sick (visual symptoms) adults with *j* adult animals from a noninfected culture in four different proportions (*i* + *j*), 6 + 0, 4 + 2, 2 + 4, and 0 + 6, and let them reproduce for 7 days at 23°C before fixing the animals for *in situ* hybridization using 22-nt specific probes specified in Table S1c. The numbers of infected animals and cells were counted manually.

Second, we used a two-step protocol to compare virus infectivity (SANTV JUv1993, JUv1551, and LEBV JUv1498) in single infections and in competition in the host, C. briggsae JU1264. We independently infected C. briggsae JU1264 animals with the tested viral preparations (i.e., JUv1993, JUv1551, and JUv1498) in three replicates. We visually assessed symptoms on 50 adult hermaphrodites and checked that, after 7 days, more than 80% of the nematodes were infected. We then mixed 6 (3 + 3, 6 + 0, and 0 + 6) sick adult animals (visual symptoms) from the originally infected populations with either of two viruses and let them reproduce for 3 days at 23°C in three replicates. For each replicate, we passed at each generation (every 3 days) 20 L4 larvae to a new plate and fixed each generation for *in situ* hybridization. The number of (co-)infected animals and cells were counted manually. (Co-)infection levels of SANTV- versus LEBV-infected animals were scored using FISH staining with SANTV RNA1 (Cal Fluor red 610) and LEBV RNA1 (Quasar 670) Biosearch probes (Table S1b). (Co-)infection levels of JUv1551- versus JUv1993-infected animals were scored using FISH staining using JUv1264/JU1551 RNA1 (CFP/ATTO425) and JUv1993 RNA1 (Texas Red) 22-nt-long probes (Table S1c).

Finally, we used a two-step protocol to perform pairwise competition assays between SANTV JUv1993 and two other SANTV variants, JUv1264 and JUv1551. We independently infected C. briggsae JU1264 animals with the tested viral preparations (i.e., JUv1993, JUv1264, and JUv1551) in three replicates. We visually assessed symptoms on 50 adult hermaphrodites and checked that after 7 days, more than 80% of the nematodes were infected. We then mixed 20 (10 + 10) sick adult animals (visual symptoms) from the originally infected populations with either of two viruses and let them reproduce for 3 days at 23°C in three replicates. For each replicate, we passed at each generation (every 3 days) 20 L4 larvae to a new plate and fixed each generation for *in situ* hybridization using 22-nt specific probes specified in Table S1c. The numbers of (co-)infected animals and cells were counted manually.

A third step was added to the above-described experiment. We prepared a virus filtrate in the first generation of coinfection and reinfected several C. briggsae wild isolates (JU1264, JU1498, JU1993, and JU2160). We then passed at each generation (every 3 days) 20 L4 larvae to a new plate and fixed each generation for *in situ* hybridization using 22-nt specific probes specified in Table S1c.

### Data availability.

Sequences were deposited in GenBank under accession numbers MK774657 to MK774660 and MN130962 to MN131047.

## Supplementary Material

Supplemental file 1

Supplemental file 2

Supplemental file 3
